# Personalized risk predictor for acute cellular rejection in lung transplant using soluble CD31

**DOI:** 10.1038/s41598-022-21070-1

**Published:** 2022-10-21

**Authors:** Alexy Tran-Dinh, Quentin Laurent, Guillaume Even, Sébastien Tanaka, Brice Lortat-Jacob, Yves Castier, Hervé Mal, Jonathan Messika, Pierre Mordant, Antonino Nicoletti, Philippe Montravers, Giuseppina Caligiuri, Ian Morilla

**Affiliations:** 1 Département d’Anesthésie-Réanimation, AP-HP, Hôpital Bichat Claude Bernard, Université Paris cité, Paris, France; 2 LVTS, Inserm U1148, Université Paris cité, 75018 Paris, France; 3 UMR 1188, INSERM, Université de la Réunion, Saint-Denis de la Réunion, France; 4 Service de Chirurgie Thoracique, Vasculaire et Transplantation Pulmonaire, AP-HP, Hôpital Bichat Claude Bernard, Université Paris cité, Paris, France; 5 INSERM UMR 1152-ANR10-LABX-17, Paris, France; 6 Pneumologie B et Transplantation Pulmonaire, AP-HP, Hôpital Bichat Claude Bernard, Université Paris cité, Paris, France; 7 Paris Transplant Group, Paris, France; 8 LAGA, CNRS, UMR 7539, Laboratoire d’excellence Inflamex, Université Sorbonne Paris Nord, 93430 Villetaneuse, France

**Keywords:** Allotransplantation, Scientific data, Statistics

## Abstract

We evaluated the contribution of artificial intelligence in predicting the risk of acute cellular rejection (ACR) using early plasma levels of soluble CD31 (sCD31) in combination with recipient haematosis, which was measured by the ratio of arterial oxygen partial pressure to fractional oxygen inspired (PaO_2_/FiO_2_) and respiratory SOFA (Sequential Organ Failure Assessment) within 3 days of lung transplantation (LTx). CD31 is expressed on endothelial cells, leukocytes and platelets and acts as a “peace-maker” at the blood/vessel interface. Upon nonspecific activation, CD31 can be cleaved, released, and detected in the plasma (sCD31). The study included 40 lung transplant recipients, seven (17.5%) of whom experienced ACR. We modelled the plasma levels of sCD31 as a nonlinear dependent variable of the PaO_2_/FiO_2_ and respiratory SOFA over time using multivariate and multimodal models. A deep convolutional network classified the time series models of each individual associated with the risk of ACR to each individual in the cohort.

## Introduction

Lung transplantation (LTx) is a life-saving therapy that may be offered to selected patients with end-stage lung disease. However, lung transplant recipients have the lowest survival rate among solid organ transplants of 6.7 years^1^. Indeed, their prognosis can be hampered by numerous complications, including primary graft dysfunction^2^, infections^3^, airway complications^4^ and acute rejection^5^. Despite advances in immunosuppressive treatment strategies, 30–50% of recipients experience at least one episode of acute cellular rejection (ACR) during the first year after LTx^6,7^. ACR is triggered by a T lymphocyte-induced response to allogeneic human leucocyte antigen or other antigens^8,9^, which is characterized by the infiltration of mononuclear cells around the pulmonary capillaries and/or small airways^10^ that target and damage the graft tissue. ACR is a major risk factor for chronic lung allograft dysfunction, which is the leading cause of mortality beyond 1 year of LTx and accounts for over 25% of deaths^11^. In a multicentre, prospective study, Todd et al. reported that more than 50% of LTx recipients experienced at least one episode of ACR within the first year, with a median delay of 43 (27–100) days^7^. The diagnosis of ACR is suspected in the presence of respiratory deterioration but remains very challenging. Indeed, ACR has no clinical, radiological, or biological specificities with respect to other causes of decreased lung function^12^. Moreover, ACR can occur in asymptomatic patients or those who develop nonspecific symptoms such as cough, fever, or flu-like manifestations^13,14^. Once suspected, the diagnosis of ACR is confirmed by histopathological evidence on transbronchial lung biopsies^15–17^. However, there are no recommendations on how often and how to perform transbronchial lung biopsies^15^. In addition, the anatomopathological interpretation of acute rejection suffers from a high degree of inconsistency, partly due to the inter-observer variability^18^.

ACR results from the initial activation and interaction of graft endothelial cells with lymphocytes, neutrophils and platelets. Allogeneic presentation by graft endothelial cells is a primary target for circulating T cells^19,20^, and the association of endothelial activation with acute rejection has been previously observed in heart transplantation^21^. The role of platelets in acute rejection has been studied in kidney^22^, cardiac^23^ and skin allograft^24^. After the initiation of allograft rejection by T cells, platelets help recruit T cells and increase the plasma inflammatory mediators, which accelerates the T-cell-induced rejection^24^. Neutrophils also play an important role in the acute rejection of solid organ transplants^25^.

The development of surrogate biomarkers with the assistance of artificial intelligence technology to identify recipients at risk of developing ACR is a major diagnostic aid to the clinician^26^.

We hypothesized that the severity of endothelial dysfunction and impaired haematosis assessed very early after LTx might be associated with the occurrence of ACR.

CD31 is a promising biomarker of acute rejection. CD31 is a 130-kDa glycosylated transmembrane immunoglobulin-type inhibitory receptor that is constitutively and exclusively expressed on endothelial cells, leucocytes (including T- and B-lymphocytes, dendritic cells, neutrophil monocytes and macrophages)^27^, and platelets^28,29^. CD31 is involved in maintaining homeostasis at the blood/vessel interface^30^. CD31 is composed of an extracellular domain comprising 6 Ig-like domains numbered from 6 to 1 from the membrane part to the most distal extracellular part, a trans-membrane segment and a cytoplasmic tail containing 2 immuno tyrosine-based inhibitory motifs (ITIMs)^31^. CD31 molecules on interacting cells bind to each other via a trans-homophilic interaction of extracellular domain 1 that triggers protein clustering via a cis-homophilic interaction of the extracellular juxta-membrane sequences; thus, they promote the phosphorylation of the intracellular ITIMs of CD31 by tyrosine kinases. The phosphorylation of ITIMs triggers the recruitment and activation of SH2-containing phosphatase signalling pathways, which leads to an inhibitory effect on tyrosine kinase-dependent cellular functions and an activating effect on SH2 phosphatase-dependent functions. CD31 is involved in inhibiting the reactive oxygen species formation^32^ and inflammatory signalling by ICAM-1^33^ and IL-1β^34^. In addition, CD31-mediated signalling is required for cell survival^35^, prostacyclin release^36^, regulation of arteriolar tone^37^, barrier integrity^38^ and angiogenesis^39^. Upon cell activation, regardless of the stimulus, the extracellular portion of CD31 is enzymatically cleaved and released from the cell surface. The shed CD31 can be detected in the plasma in a soluble form (sCD31)^40–45^. Truncated CD31, which remains anchored on the cell membrane, loses its primary function as a "peacemaker" and contributes to enhance the cell activation. We hypothesized that the sCD31 released from activated endothelial cells, platelets and leukocytes and detected in blood samples of lung transplant patients could be used as a predictive biomarker of ACR.

The ratio of arterial oxygen partial pressure to fractional inspired oxygen (PaO_2_/FiO_2_) is used to assess the haematosis of the lungs and classify the severity of acute respiratory distress syndrome^46^. Measurement of the PaO_2_/FiO_2_ ratio 24 h after LTx has been shown to correlate with mortality^47^.

In this study, we provide a systemic model that can connect the plasma levels of sCD31 to the PaO_2_/FiO_2_ ratio over time. The model unveils a behavioural pattern that can be used early on to stratify patients who suffer episodes of ACR using multivariate and multimodal models of time series^48,49^. Then, facing more limited traditional statistical models^50,51^, we evaluated the contribution of machine learning (see Fig. S0) in the prediction of the risk of ACR after LTx. With this goal in mind, we constructed a deep convolutional network^52^ that classified the time series models of each individual. Thus, we adjusted the parameters derived from every patient’s outcome modelled as a time series and improved their initial guesses. Our model successfully utilized the scarce available information. Additionally, we avoided the inadequate consequences of learning from datasets with large label imbalances due to the calibration of the trained convolutional network by weighting the patient outcomes^53^. Then, we encoded the pre-activation of the penultimate layer with a similar procedure to that used by the log-odds^54^ to build a risk predictor of ACR. Finally, this predictor was associated with a percentage of accuracy for all patients in the cohort.

## Results

### Demographic data and patient outcomes after lung transplantation

Forty patients were included in the study between December 2016 and February 2021. The lung transplant recipients were aged 60 (52–64) years and mainly of the male sex (70%). They were transplanted for emphysema (33%), interstitial lung disease (50%) or other aetiologies (17.5%). They received single (45%) or double LTx (55%). After LTx, patients had a median (IQR) length of stay in the intensive care unit (ICU) of 19 (13–39) days and were mechanically ventilated for a median (IQR) time of 2.5 (1–6) days. The mortality rates in the ICU and at 1 year were 5% and 15%, respectively. Seven (17.5%) patients had at least one episode of ACR within 1 year after LTx, with a median (IQR) time to onset of 18 (13–221) days. Five ACR were classified as A1 and two as A2. The median (IQR) postoperative plasma sCD31 levels were 4240 (2753–6114) pg/ml at H24, 4251 (2860–6197) pg/ml at H48 and 4285 (2950–6414) pg/ml at H72. Patients with and without ACR had median (IQR) plasma sCD31 levels of 4280 (3137–4646) and 4160 (2738–6428) pg/ml at H24, 3757 (2570–4173) and 4618 (3184–7105) pg/ml at H48, and 3259 (2753–6154) and 4773 (3099–6871) pg/ml at H72, respectively. The mean sCD31 levels of patients with ACR grades A1 and A2 were 3973 and 12,688 pg/ml at H24, 2826 and 5799 pg/ml at H48 and 3597 and 2596 pg/ml at H72, respectively.

### Establishing the systematic view of sCD31 as a biomarker of reference during patient post-operational tracking

To better understand the potential of sCD31 in predicting ACR after LTx, all empirical data observed by clinicians during the post-operation period had to be formalized. Since rehabilitation tracking is a dynamic process over time, the measured plasma levels of sCD31 were treated as a time series. We approached a varied set of classical machine learning methods to complete this task. Initially, univariate time series modelling of sCD31 based on linear regression with overlaying paddings enabled us to predict the patient outcomes^55,56^. Using totally random tree models^57^ and unsupervised hierarchical machine learning, we obtained the best percentage of success in patient stratification, reaching 78% (see supplemental material and Fig. S1). This result confirms that sCD31 is a critical checkpoint for ACR complications. However, this approach missed many more false negatives than expected, potentially due to the total recall achieved by the univariate model, which was 0.70–0.83. This recall range was not sufficiently high for a cohort displaying these characteristics (see “Methods”). Thus, the univariate model did not properly reflect the physiological power of sCD31. Moreover, the spectral plots of the stationary series could not be used to discriminate between non-ACR and ACR patients.

### The inclusion of the PaO_2_/FiO_2_ ratio largely ameliorates the prior univariate model reinforcing sCD31 as a unique physiological spot

The measured PaO_2_/FiO_2_ exchange ratio is an important parameter when quantifying the effects of therapeutic interventions or when specifying diagnostic criteria for acute lung injury and acute respiratory distress syndrome^58^.

**Figure 1:**
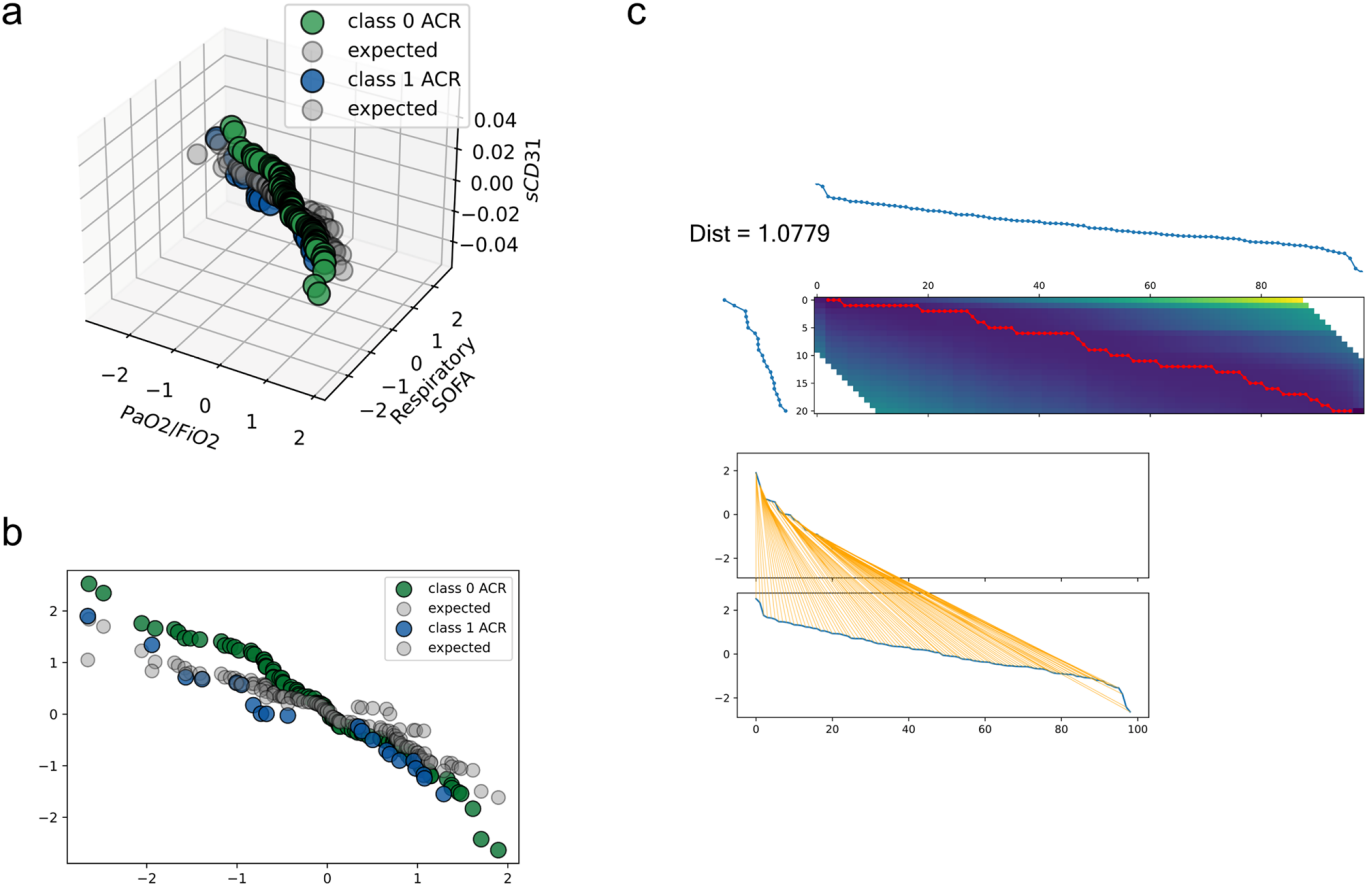
Remarkably higher sCD31 concentration in patients who experienced ACR after LT. A relative concentration of sCD31 to ACR might be observed in (**a**). Whether or not the patients experienced any complication was highlighted in green or blue in the curves. The expected value predicted by our VAR model is shown in grey. A 2D projection of (**a**) is shown in panel (**b**). The grade of separation in sCD31 concentration between patients with or without complications after LT was calculated using 2d-DTW (see “Methods”) in upper panel (**c**). The distance function has linear space complexity but quadratic time complexity. Panel c displays the matrix with all possible warping paths (i.e., accumulated cost matrix) that resulted in a perfect match between indices of ACR time series.

Overall, the integration of the above in our model improved the multivariate analysis of our time series model^59^. Indeed, such analysis outperformed the univariate modelling of sCD31 in the previous section. The spectral analysis on both biomarkers as time series revealed a hidden bidirectional relationship between series^60^. In particular, the quantitative behaviour of sCD31 plasma levels over time was influenced by PaO_2_/FiO_2_ (see Fig. 1). Furthermore, sCD31 as a time series could be fully explained in terms of PaO_2_/FiO_2_ as an absolute value ratio and respiratory SOFA (Sequential Organ Failure Assessment) using a multivariable function, which followed a vector autoregression model (VAR)^59,61^. Then, the predictors were only the lags of sCD31 and PaO_2_/FiO_2_ series (see “Methods”).

At first glance, the series has a similar trend profile over time tags (i.e., 24, 48, and 72 h post-transplantation), except for PaO_2_/FiO_2_, where a slightly different profile of anti-correlation is evidenced in its amplitude (see Figs. S2a-b). The Granger’s causality test^62^ to check for PaO_2_/FiO_2_ causing sCD31 yielded a *p* value of 0.0054. However, the *p* value of sCD31 causing PaO2/FiO2 was 0.4005 (see Table S1 for *p* values associated with all possible combinations of series). In both cases, the *p* values were far from the standard threshold of 0.05. Consequently, the null hypothesis (see “Methods”) that stated that the coefficients were equal to zero in the autoregression process could be rejected.

Now, if a set of time series can be co-integrated^63^, they have a long-term, statistically significant relationship. Regarding our biomarker series, respiratory SOFA could not be co-integrated into the other two series before starting to build the VAR model. The associated statistic of respiratory SOFA lay below the bound limit identified by the cointegration test as valid (see “Methods”); this value corresponded to 4.1296 with a confidence interval of 95%.

Next, we want our selected statistical features not to vary over time, since it is a necessary condition to build the VAR model. In brief, we fitted the VAR model on a training dataset derived from the sCD31 data, and we used the trained model to forecast the next *k* observations (training, test = data[0:-k], data[-k:]). The augmented Dickey–Fuller^63^ with only differencing (see Table S2) made the trick pass the respiratory SOFA *p* value from 0.408 to 0.0001. Finally, actual and expected forecasts will be crossed during the testing task against those in the test set. The statistical goodness of fit of the model is approached using multiple forecast accuracy metrics such as root-mean-square error (RMSE) and mean absolute percentage error (M(A)PE).

To select the right lag order of the VAR model (see Supplemental material), we iteratively fitted increasing orders of the model. Then, we picked the order associated with the VAR model with the least Hannan-Quinn information criterion (HQIC) (see Table S3 for other tested scores). The lowest HQIC resulted at lag 3, so we trained our VAR model using that order. No serial correlation was observed in the model since the Durbin-Watson statistic (DW) remained close to 2 across the series (for further details, see supplemental material and Table S5)^64^. Finally, we could generate the forecast of the testing data for sCD31, PaO_2_/FiO_2_, and respiratory SOFA series, whose computing scaled the magnitude to the training data used by the model. Therefore, to bring it back up to its initial scale, we de-differenced once as noted above. The accuracy of the resulting VAR model is provided in Table S6 and Fig. S2c.

To the best of our knowledge, this is the first time that a functional influence of this type has been established in the post-operative follow-up of a pulmonary transplant. We did not claim the PaO_2_/FiO_2_ ratio to be an important physiological player in predicting the risk of ACR, but it is relevant for the stability of our VAR model of sCD31.

### The modelling of sCD31 as a multivariate time series provides strong evidence of its post-transplantation predictive power

After the autoregression process, we set a decreasing order for the values of the series derived from the VAR model, which synchronised the time series profiles forecasted from the model. Surprisingly, when we plotted the model predictions for the testing data, they slammed down as expected, and they preserved a unique pattern in accordance with rejected or accepted recipients across patients of the cohort. Thus, the sCD31 vs. PaO_2_/FiO_2_ profile modelling plasma levels and haematosis over time displayed by patients in whom the surgery failed sharply dropped in those patients who were categorized as definitely accepting the transplantation (Fig. 1a,b). It was reasonable to consider it a potential good criterion to apply to differentially stratify recipients early on. Consequently, this result confirms the powerful discriminant role that our VAR model assigns sCD31 during post-operational patient tracking.

Using dynamic time warping (DTW)^65^, we quantified (see “Methods”) the decrease in sCD31 plasma levels exhibited in the series forecasted from our model. We measured the distance between two dependent 2-dimensional sequences with $\left({\mathbb{R}}^{T\times P}\right.{)}^{3k}$ time steps (see “Methods”). The first dimension of the data was assumed to be the time series index. With this calculation, we qualitatively compared the temporal series per class and the complication. Thus, the normalized distance between non-ACR and ACR patients was 1.0779 (see Fig. 1c). This result indicates that an accurate qualitative distinction has been achieved between classes of patients with ACR complications (see Fig. S3).

### A deep neural network classifier of the sCD31 time series accurately predicts the outcomes of patients

**Figure 2:**
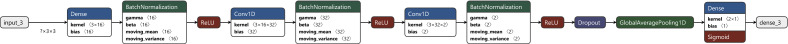
Architecture of our temporal network. Detailed design of our learning model for the feature extractor to CD31 channels when the time series takes a length of $3P$. We used a similar schema for PaO_2_/FiO_2_ channels. If an amalgamation of both sCD31 and PaO_2_/FiO_2_ and respiratory SOFA is considered, the outputs of the dropout layers are concatenated and fed into the final classifier. Thus, the last dense layer of our temporal network is composed of a set of cardinal parameters equal to $3\times (($T + T′)$\times (3P//32)\times 2)$.

During training, we adjusted the parameters of our model to improve the guess about the input of the VAR models. Our strategy does not rely on a priori calculated derivation, but it benefits from the model processing of multivariate time series. Thus, the proposed model learns based on the underlying nonlinear behaviour of sCD31 plasma levels as a time series to be classified from multivariate and multimodal models. Then, we constructed a machine learning architecture (see Fig. 2), which preserved this premise. The time series fed into the learning model were additionally standardized to ensure the existence of an adequate probability space where to generate of our ACR risk predictor. This predictor largely ameliorated the impact of the spectral patient stratification in the previous section. In particular, with a smart design of convolutional neural network (CNN) feature extractors^66^, which consisted of parallel processing of different series modalities using a linearly activated dense input layer and two convolutional 1D (conv1d) layers, all three passed by batch normalization layers that were nonlinearly activated with 64 (64) + 1568 (128) + 194 (8) trainable parameters each, the resulting tensor passed through a dropout layer that randomly removed 25% of outgoing neurons to reduce overfitting, and the global average pooling1d layer was averaged among all time steps of the pool sizes. A final dense layer with a sigmoid activation function with 3 trainable parameters formed the actual time series classifier (see “Methods”). Ultimately, we implemented a personalized risk predictor of everyone experiencing an ACR in the cohort based on the cumulative probability distribution associated with the pre-activation values of the penultimate layer.

**Figure 3:**
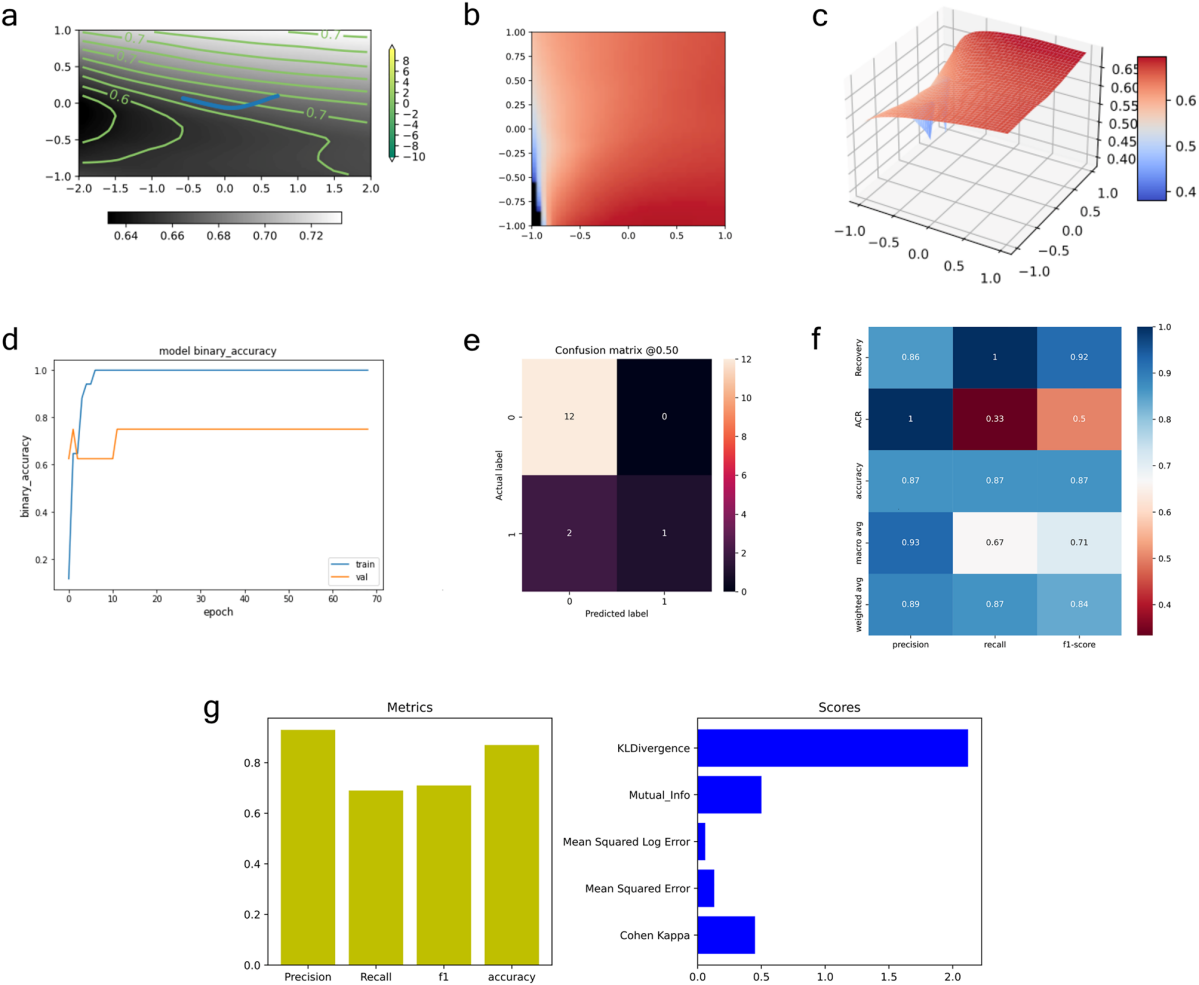
Results from the initial bias corrected and weighted temporal network. (**a**–**c**) Trainable parameter landscapes of the deep temporal network. The plots show the learning progression of our model across epochs and batches. (**a**) Contour with optimal projected trajectories in blue in the [− 1, 1] range. Those trajectories go from “mountain” values near 1 to a “valley” near − 0.73. From the green levels’ density and blue maps, we can confirm 1 and − 0.73 as the global coordinates to be travelled by the network to arrive in good convergence. (**b**) Grid projection exhibiting a global maximum collapsing in 1 and a stretcher option for “valleys”. (**c**) Surface composed of the required weights to achieve the optimal sought by the Adam algorithm, which is scaled by their z scores. (**d**) Training history of the corrected bias initialization and weighted model. We visualize accuracy across the chosen number of epochs and batches. A global quick look appears to show a standard training scenario with minor issues concerning the validation results in precision and recall panels at the beginning of the training task. (**e**) Goodness of fit of our weighted deep network during the training task. We calculated its confusion matrix where we could observe only two misclassified patients. However, we accurately detected false negatives compared to the baseline model. (**f**) Final heatmap associated with the metrics in the learning process of our weighted model. The balance among accuracy, recall and precision values is optimal to prevent missing false negatives. (**g**) Assessment of our model during the testing validation of our weighted model. We plotted it in terms of the average metrics (left) and scores of learning performance (right) on *Cohen Kappa*, *Kullback Leibler* divergence (KL Divergence) or Mean Squared Error. The formers are especially useful because we had imbalanced data. The KL divergence describes the inefficiency caused by the approximation of the true target distribution with the expected target distribution. KLDivergence does not have an upper bound; thus, a score of approximately 2.0 can be interpreted as a moderate or fair measure of how our model estimates the true target distribution.

Starting from a baseline model (see Fig. S5), we applied an initial bias correction to partially improve the training performance of the model with the raw series. The baseline model reported a fair classification of ACR patients but with a difficult interpretation of the precision and recall values returned during the training task. The baseline model resulted in a loss of 0.46–0.66, an average accuracy of 75% and f1-scores, recall and precision of 0.33. This was possibly due to an imbalance in the class dataset. Thus, the model was recalibrated using class weights. Here, with the class weights, the average accuracy and precision were 0.87 and 0.93 per class, respectively, with a high ratio of false positives. Simultaneously, the recall (1 and 0.33) and area under the curve (AUC = 0.85) reached their best values because the model found more true positives than the baseline model. The weighted model achieved its maximal accuracy and recall and identified more false negatives (see Fig. 3). We additionally tested f1 and Cohen’s kappa scores^67^, which are especially useful (as opposed to accuracy metrics) in the presence of imbalanced classes (see “Methods”). The f1-score that combines precision and recall was 0.92 and 0.5 (recovery and ACR classes), respectively, whereas the associated Cohen’s kappa was in the range from good to excellent with a value of 0.44 (see “Methods”). The confusion matrix shows that on average, only two patients of the test data were missed. Finally, the learning surface corresponding to the weighted model was composed of 1929 trainable parameters (see Fig. 3a–c and Fig. S6 for further details on the regularisation of this variational manifold).

### The predictions resulting from the learning model generate a personalized risk predictor of ACR

Upon fixing the required probabilistic space by initially standardizing the series, the last dense layer of the time distributed network conformed to a valid classifier of the time series of patients. Let $y$ be the output of that last layer resulting from applying a sigmoid function to the pre-activation vector. Then, we calculated the cumulative distribution function that provided the individual risk percentages of ACR. It is important to note that the z values or *log-odds* derived from the inverse of the sigmoid were used here, which stated that z could be defined as the log of the probability of the ACR patients divided by the probability of the non-ACR patients as follows:

1 $$z=\mathrm{log}\left(\frac{y}{1-y}\right),$$

where the distribution or probability density function (pdf) of each activation ${Y{^{\prime}}}_{i}$ of the penultimate layer is a multidimensional version of the rectified Gaussian distribution. This is a hybrid (discrete–continuous) distribution with a point mass at the origin, a multivariate Gaussian in the all-positive part of the space and 0 everywhere else. Then, the z-values transform into probability due to the following conversion:

2 $$p\left( {Z = z} \right) = {\raise0.7ex\hbox{${e^{z} }$} \!\mathord{\left/ {\vphantom {{e^{z} } {(1 + e^{z} )}}}\right.\kern-\nulldelimiterspace} \!\lower0.7ex\hbox{${(1 + e^{z} )}$}},$$

Hence, we could associate a risk of ACR from the classification of each patient series based on their sCD31 plasma levels post-transplantation by:

3 $$F_{Z} \left( z \right) = p\left( {Z \le z} \right) = \mathop \smallint \limits_{0}^{z} {\raise0.7ex\hbox{${e^{t} }$} \!\mathord{\left/ {\vphantom {{e^{t} } {\left( {1 + e^{t} } \right)}}}\right.\kern-\nulldelimiterspace} \!\lower0.7ex\hbox{${\left( {1 + e^{t} } \right)}$}}dt.$$

## Discussion

In this work, we introduce a multivariate and time-distributed learning model based on the early evaluation of sCD31 cleavage in triggering ACR after LTx. The model takes advantage of other important clinical features such as PaO_2_/FiO_2_ or respiratory SOFA to extract the best guess of sCD31 from it. The outcome of each patient is compared to their expected predictions; then, we adjust the model’s parameters by training tasks to improve the guess. To the best of our knowledge, this is a *one-of-a-kind* model in the literature. Beyond the limitations of any model, our predictor bridges the gap between health care management and a field where clinicians’ standards of diagnosis are still far from being computerized.

sCD31 is a prime candidate as a biomarker to predict ACR. Indeed, sCD31 results from the cleavage and shedding of CD31 into plasma following endothelial, leukocyte and/or platelet activation, which are key features in ACR. Therefore, sCD31 is used as a reflection of altered CD31.

Several studies have shown that CD31 plays a protective role in acute rejection, since the native function of this receptor is to counteract excessive activation of the endothelium, leukocytes and platelets.

Using an in vitro model of porcine cells, human CD31 suppressed neutrophil-mediated xenogenic cytotoxicity by inhibiting NETosis^68^. Cheung et al. showed that CD31 conferred an immune privilege to the vascular endothelium, which prevented the apoptotic death of endothelial cells induced by cytotoxic T lymphocytes and TNF-α^69^. In the same study, the authors compared the rejection of male-derived wild-type and CD31-deficient skin grafts by syngeneic female recipient mice. Female mice that received CD31-deficient male-derived skin grafts had significantly reduced survival compared with those that were grafted with wild-type skin. Moreover, they showed that the transduction of the CD31 gene to CD31-deficient pancreatic β cells enabled them to recover the cytoprotective mechanisms against extrinsic apoptosis. Transplantation of CD31-transduced pancreatic β-cells into allogeneic diabetic recipient mice controlled their blood glucose levels, whereas diabetes persisted in the mice that were transplanted with CD31-deficient pancreatic β-cells. Similarly, Ma et al. showed that CD31 played a nonredundant role in the regulation of T-cell immunity and tolerance. In a skin grafting model where wild-type or CD31^−/−^ deficient female mice received wild-type male skin, HY mismatch resulted in the T-cell-mediated rejection of male skin grafts. Skin graft rejection by CD31^−/−^ females was significantly accelerated and more pronounced than that of wild-type females^70^. CD31 plays a complex role in the regulation of T-cell-mediated immune responses^71^. One of the most studied roles of CD31 is its ability to inhibit the protein tyrosine kinase-dependent signal transduction mediated by the immunoreceptor tyrosine-based activation motif-containing T-cell receptor^72^. Alloreactive memory T cells are an essential component of the allograft rejection process and a major obstacle to tolerance induction in clinical transplantation^73^. Interestingly, CD31 selectively attenuates the chemokinesis of memory and activated T cells, which helps homeostatically regulate the effector T-cell immunity^74^.

In addition, sCD31 can be used as a target for in vivo molecular imaging of ACR, since our group has shown that targeting cleaved CD31 is an attractive strategy for specific in vivo imaging of inflammatory processes^75^.

Herein, we designed a smart architecture of a deep neural network to create a personalized risk predictor of ACR based on the sCD31 plasma level classification from multimodal and multivariate time series. The proposed model pools information from three different categories of biomarkers due to a linear spatial filtering operation^76^ and builds a hierarchical feature representation of the post-operational records of a lung transplant based on temporal convolutions. It pulls complementary information over different categories processed with separate pipelines, and it optimizes at maximum all available information related to a small cohort.

Our model in this work displays robust classification performances compared to the state-of-the-art, which is lacking in lung transplant track models. There are no artificial intelligence-derived models, but few results have mainly focused on less adaptable statistical models. Conveniently, our model takes a short run time and a low computational cost. Thus, the approach is a potential good candidate for use in a local device and to perform online ACR patient prediction for clinician analysis. Our approach enables the quantification of the use of multiple sCD31 plasma levels and additional categories, such as PaO_2_/FiO_2_ and respiratory SOFA. Interestingly, our model missed very few false negatives during the analysis and maximized its accuracy. In the end, this was the best indicator of our model goodness. Furthermore, using class weights boosted the model performances. We alternatively applied LSTM^77^ with class oversampling to classify the ACR patients. For a model with 512 layers and 0.25 dropout, we obtained quantitatively similar results as CNN but with less interpretable evidence of model accuracy, conceivably due to a null recall range achieved by the model during the training task.

The most important concern related to this study may be the limited number of patients analysed. Additionally, but to a lesser extent, the reliability of making medium-term predictions based on short-term data may be further discussed. However, to the best of our knowledge, there is no systematic learning model of surrogate biomarkers to identify recipients at risk of developing ACR. In this context, the proposed approach is a novel model that charts the outcomes of traumatic solutions related to the treatment of severe pulmonary diseases. Therefore, our model based on the time-distributed analysis of a pool of precious samples should be considered a valuable tool for anyone in the domain. Complementarily, we envisage extending our work by predicting the time to the next episode of ACR.

Another limitation is that sCD31 and haematosis parameters (PaO_2_/FiO_2_ ratio and respiratory SOFA) are dysregulated in many forms of physiological stress to the lungs, such as pneumonia and primary graft dysfunction. The predictive value is lower for primary graft dysfunction (PGD) because the measurements of sCD31, PaO_2_/FiO_2_ ratio and respiratory SOFA are performed within 72 h after LTx, which is also the time of onset of PGD in its definition. However, the specificity of the model that we use to predict the occurrence of ACR versus the occurrence of other respiratory complications should be assessed in a future study.

We minimized the effect of a “low number of samples (n = 40)” by leveraging a linear spatial filtering layer earlier on the deep network. The introduction of on-bottom frozen layers of samples resulting from linear combinations, such as that occurring in transfer learning, helped robustly train our full-scale model from scratch. The latter might be dissipated by considering a multimodal and multivariate temporal network at the base of our learning model. That structure enabled the accurate forecasting of our predictions in the medium term. Another interesting approach may be to spot the best stride between actual and forecasted time series and predict only there to prevent the model from predicting the entire series.

In conclusion, our analysis demonstrates the benefit of using the temporal context with training tasks in ACR prediction using sCD31, which is a unique biomarker that can reflect endothelial, leukocyte, and platelet activation. Furthermore, its quantification appears to significantly increase the performance when the number of biomarkers is limited. In large cohorts with a larger number of associated biomarkers, the use of temporal context as proposed here for online prediction may require adjustments. However, the flexibility of our model enables clinicians or anyone in the field to easily use it for prediction on local servers.

## Methods

### Design

This is an interventional, prospective, and single-centre study conducted from December 2016 to December 2018. The study was approved by an Institutional Review Board (French national ethics committee for the protection of persons undergoing research, “Comité de Protection des Personnes Sud-Est III”, number 2017-054 B), which confirmed that the study met the validation conditions set out in Article L. 1123.7 of the Public Health Code. All participants provided written informed consent. All methods were performed in accordance with relevant guidelines and regulations.

### Determination of the plasma sCD31 concentration and PaO_2_/FiO_2_ ratio

For each patient, blood samples were collected at 24, 48 and 72 h after LTx, centrifuged twice at 2500*g* for 15 min and frozen at − 80 °C. Endothelial shed CD31 was assessed by incubating 50 µl of plasma sample with functional cytometric beads coupled to WM59 monoclonal purified antibodies directed against human CD31 domain 1 (Thermo Fischer Scientific). Positive binding of sCD31 was detected by the anti-CD31 monoclonal antibody MBC78.2 directed against human CD31 domain 6 (Thermo Fischer Scientific). The standard curves were obtained with each detecting monoclonal antibody that was simultaneously used with recombinant CD31 to overcome any bias due to differences in binding affinity of the diverse antibodies. Analyses were performed using the Bio-Plex® 200 system. The PaO_2_/FiO_2_ ratios were calculated from the arterial blood gas analysis, which was simultaneously taken with the sCD31 samples.

### Diagnosis of acute cellular rejection

ACR was suspected to occur in the year following LTx when there was a combination of clinical and radiological evidence. Transbronchial lung biopsy was performed for anatomopathological analysis to confirm or deny the suspicion of ACR. ACR was graded as previously defined based on the perivascular and interstitial mononuclear infiltrates of the lung allograft^10^.

### Adequate notation

We denote by *X*∈ ${\mathbb{R}}^{T\times P}$ a segment of exogenous time series with its label $\mathcalligra{y}\in \mathcal{Y}$ that maps to the set {*A, R*$\}$, where *A* denotes accepted and *R* denotes rejected. Here, *X* corresponds to a sample record, and $\mathcal{Y}=\left\{\mathcalligra{y}\right.\in {\mathbb{R}}_{+}^{2} : \sum_{i=1}^{2}{\mathcalligra{y}}_{i}=\left.1\right\}$ corresponds to the probability convex simplex. Specifically, each label is encoded as a vector of ${\mathbb{R}}^{2}$ with one coefficient equal to 0 and a single coefficient equal to 1, which indicates the post-operational stage. Here, *T* is the number of time points* P* and refers to the number of samples. ${\mathcal{T}}_{t}^{k}=\left\{{X}_{t-k},\ldots ,{ X}_{t}, \cdots , {X}_{t+k}\right\}$ is a sequence of 3$k$ variables per time point. ${\chi }_{k}=\left({\mathbb{R}}^{T\times P}\right.{)}^{3k}$ determines the space of the $3k$ consecutive multivariate time series. Finally, ${\mathcal{B}}_{\mathcalligra{l}}$ is the binary cross entropy loss function. Given a true label $\mathcalligra{y}\in \mathcal{Y}$ and a predicted label $p\in \mathcal{Y}$, it is defined as ${\mathcal{B}}_{\mathcalligra{l}}=-\frac{1}{2}\sum_{i=1}^{2}{y}_{i}\mathrm{log}{p}_{i}$.

### sCD31 and PaO_2_/FiO_2_as time series models

Similar to an electrocardiogram measuring the heartbeat pace of patients to diagnose heart disorders, our approach transforms the problem of tracking sCD31 activity in plasma into an analytic matter of time series. We pre-processed the raw dataset to be adequately interpreted by a multivariable model. This task consisted of determining whether sCD31 and PaO_2_/FiO_2_, which are considered variables, could influence each other depending on their past values. To prove this hypothesis, we reshaped the original ${\mathbb{R}}^{9\times 40}$ matrix as an ${({\mathbb{R}}}^{3\times 3}{)}^{40}$ one. This is the dimension of the transformed space of latent features ready to be predicted using multimodal models in the classification of time series datasets. Additionally, we set the respiratory SOFA (Sequential Organ Failure Assessment) time series, which is an invasive arterial monitoring tool to measure the arterial partial pressure of oxygen and subsequently calculate the PaO_2_/FiO_2_ ratio^78^. This discrete clinical indicator acts as a “categorical PaO_2_/FiO_2_ feature” with important implications in the assessment of the acute morbidity of critical illness^78^. Before the classification task, we used the dynamic time warping (DTW)^79^ to align the time series and calculate their distances between classes.

### Machine learning problem

In this section, we translate the classification of patient time series into the formal language of mathematics. Thus, for every nonnegative integer $k,$, we define the predictive model $J:{\mathcal{X}}_{k}\to \mathcal{Y}.$ Each model is in a parametric set $\mathcal{M}$. Model $J$ applies an ordered sequence of $3k$ consecutive intervals of patient time series to a probability vector $p\in \mathcal{Y}$. For simplicity, we do not show the network parameterisation. Hence, the machine learning problem can be written as follows:

4 $$\widehat{J}=arg\underset{J\in \mathcal{M}}{\mathit{min}}{\mathbb{E}}_{x,y\in \mathcal{X}\times \mathcal{Y}}\left[{\mathcal{B}}_{\mathcalligra{l}}\left(J\left(x\right),y\right)\right]$$

Equation (4) optimizes the parameters of the neural network $J$ by minimizing the expected value of the binary cross entropy between the output of this network $J(x)$ and the true label $y$.

### Multivariate network design

To stratify the evolution of patients after lung transplant, we designed a deep network that consisted of three main features: linear spatial filtering to estimate linear combinations of matrices from patient variables as transfer learning does^80^, convolutive layers to acquire spectral features and separate routes depending on the time variables sCD31 and PaO_2_/FiO_2_, and respiratory SOFA (see Fig. S4). This network traces the global feature extractor herein defined by ${F}_{t }: {\mathbb{R}}^{T\times P}\to {\mathbb{R}}^{D}$, where D is the dimension of the estimated feature space. Our network supports multiple combinations of input variables from patients and several categorical metrics simultaneously (see Fig. S4 for its on-top visualization). Multivariable models of sCD31 based on PaO_2_/FiO_2_ (respiratory SOFA) causality are learned using the vector of autoregression model (VAR). Later, these feature structures will be standardized and used as features of a multimodal deep convolutional network^81^.

Next, we explained in depth different parts of the network (for further details, see Fig. 2). We could find a primary layer that performed a time-independent linear operation to obtain a set of mixed time series, each of which resulted from a linear combination of the initial input variables. Then, it implemented a spatial filtering driven by the classification task to be executed. In particular, the usage of an appropriate 1D convolution with kernel of dimension (T,3) and a dense layer on top ensured the completion of a first layer driven in terms of spatial filters (see layer 3 in Fig. 2).

Upon the implementation of that first linear operation, we stacked two more convolution layers followed by nonlinearity and max pooling. The parameters were fixed for sCD31 plasma levels sampled at 24, 48, and 72 h. In this case, the number of time tags was $T=3\times 40=120$. Each block first minimally convolved its input signal, maintained the size of each filter as 1 × 3 but steeply increased to 32 and decreased to 2 total learned filters with stride 1 (amounts to 24 h) before applying a rectified linear unit, i.e., the so-called ReLU nonlinearity of expression $x \mapsto {\text{max}}\left( {x,0} \right)$^82^. Then, the output size was reduced along the time axis using a global average pooling 1D layer (size of 3 without overlay). Finally, we entered the output of these two convolutional blocks in a batch normalization layer^83^, which randomly selected 25% of its outward neurons and put their updates in the background at each gradient step.

The sCD31 and PaO_2_/FiO_2_ time series were treated together, since their types were comparable in magnitude, and both measured similar signals, i.e., passive diffusion of soluble molecular biomarker plasma levels. This smart and recurrent idea has been used by practitioners who approach problems of other domains, such as sleeping stage classification. In that case, series with signals of similar order are kept in methodological decomposition to better reject particular series artefacts^48^. The respiratory SOFA time series, which have different statistical and spectral properties, are processed in a parallel pipeline.

Then, we created the feature space of dimension D by combining the resulting outputs. This asset-backed space was fed into the ending layer, which was endowed with 2 neurons and a sigmoid nonlinearity to obtain a probability vector that summed to 1. This final layer is called a sigmoid classifier^84^. If we consider $a\in {\mathbb{R}}^{2}$ as the pre-activation of the last layer, the output of the network is a vector $p\in \mathcal{Y}$. Hence, *p* was determined as follows: ${p}_{i}=\frac{1}{1+{e}^{-\sum_{j=1}^{2}{a}_{j}}}$.

We also weighted patient classes to prevent the classifier from being affected by the bias derived from the label’s imbalance. The class weights were injected during the training model to minimize the range of loss.

### Time distributed multivariate network

In a VAR model, each variable is a linear function of its past values and the past values of all other variables. Thus, the predictors will be grounded on their lag values of the series. Thus, we leveraged VAR to quantify the bidirectional influence of sCD31, PaO_2_/FiO_2_ ratio and respiratory SOFA on each other. Early on, the Granger causality test of all possible time series combinations provided us with the *p* values that had to reject the null hypothesis that the coefficients of past values in the regression equation were zero. In simpler terms, the past values of PaO_2_/FiO_2_ time series do not cause the sCD31 series. Moreover, we co-integrated those time series whose linear combination ranked below their single components when the series became stationary (i.e., the mean and variance did not change over time). To this end, we performed the augmented Dickey–Fuller test on sCD31 and PaO_2_/FiO_2_ series. However, only a difference per patient was required to make the series stationaries^85^. Then, the VAR model was constructed by selecting its right lag order as a function of the Hannan-Quinn information criterion (HQIC) score^86^. For a given time series, the matrix form of the algorithm can be written as follows:

5 $$\left[Y\right]=\left[{a}_{1}\right]+\left[{W}_{1}\right]\left[{Y}_{1}(t-1)\right]+\cdots +\left[{W}_{p}\right]\left[{Y}_{1}\left(t-p\right)\right]+\left[e\right]$$

where ${a}_{i}$ are the constant terms, ${W}_{j}$ are the coefficients, and vector $e$ amounts to multivariate white noise^87^ with expected value 0 for a single series and the standard deviation of the series otherwise.

After the model had been implemented, we applied Durbin-Watson test on the residuals to evaluate the error correlations. Therefore, checking for serial correlation ensures that the model can sufficiently explain the variances and patterns in the time series. A closer value to 2 indicates that there is no significant serial correlation.

Finally, a set of a few metrics was used to check the accuracy of our model, such as the RMSE, correlation or the MAPE. The entire analysis was implemented in Python using *Statsmodels*^88^.

### Training the model

We minimize the expression in (4) using an in-house procedure based on the stochastic gradient descent setting mini batches of data. How to discriminate underrepresented classes (i.e., the ACR), and since we are interested in optimizing the balanced accuracy, we propose balancing the distribution of each class in minibatches of size 32. Because we have 2 classes, during training, each batch has approximately 5% of the samples of each class. The Adam optimizer^89^ was used for optimization with its default parameters, i.e., $lr=1{e}^{-4}$ (learning rate), ${\beta }_{1}=$ 0.9, ${\beta }_{2}=0.999$ and $\varepsilon =1{e}^{-7}$. An *early stopping call-back* on the validation loss with patience of 10 epochs was used to stop the training process when no improvements were detected. The tracking of the loss function during training initialized weights using a normal distribution with mean $\mu$=0 and standard deviation $\sigma =0.1$. Simulated linear combinations of the 40 patient matrices were also included at this stage to reinforce model optimization and cross-validated test performances (e.g., transfer learning). Since our dataset was imbalanced, we used the k-fold stratified cross-validation^90^. During cross-validation, we maintained the same class distribution in each subset. This is known as stratified sampling, where the actual classes or targets must control the sampling procedure. For example, the application from the background of a default fivefold cross-validation task (i.e., ~ 30% of training as validation data) to our model will reinforce the class distribution of each individual data batch. Thus, we ensure correspondence with the entire distribution of the training task.

Since the initial guesses were not expected to be good due to the imbalanced dataset, we set the output layer's bias^91^, which improved the initial convergence. Specifically, the bias was updated according to the expression ${log}_{e}\left(\frac{pos}{neg}\right).$ where $pos\left(neg\right)$ is the number of positive classes (resp. neg). Upon resetting the initial bias, the model returned much more reasonable initial guesses in terms of validation loss. Indeed, the fact that unlikely positive classes were not considered during the first epochs sped up its learning pace. Thus, the interpretation of the loss curves was made easier during the training history check. To ensure that we fairly compared different training runs, we applied the initial model’s weights to each model prior to the training. Next, we evaluated the loss, accuracy, precision, recall and area under the curve (AUC) metrics as measures of our model goodness. One might want to summarize the actual vs. predicted classes using the confusion matrix. We also measured the scores derived from a combination of previous metrics, such as the f1-score or Cohen’s kappa, which are especially useful in performances with label imbalance (unlike accuracy metrics). Finally, we plotted the receiver operating characteristic (ROC) curves of the test samples.

There is no ideal method to maximize both precision and recall, particularly because the classifiers handle imbalanced datasets. At this stage of the analysis, the goal is to spot false negatives (an ACR patient is missed). However, due to the limited number of ACR patients, we wanted the classifier to heavily weight the few available samples. Therefore, we passed the weights of each class through a specific parameter that was applied during the training task. Thus, we made the model focus on those samples with an underrepresented class. We retrained and evaluated the model with the class weights. Alternatively, we resampled the dataset by oversampling the minority class and reran the models. Finally, we indicate that our implementation was deployed in *Keras*^92^ with a *TensorFlow* backend^93^.

### Building the risk predictor of ACR

The individual risk predictor of ACR^94^ is founded on the training of the time distributed network, which is divided into phases I and II. The former consists of the global training of the multivariate network, notably its feature extractor part ${F}_{t}$. Then, we set the weights of the feature extractor distributed in time according to the trained model. In the second phase, those weights remain in a latent state while we train the final sigmoid classifier with aggregated features. Finally, from the evaluation of the training model using test samples, we extract the output of the penultimate layer to construct the probability distribution per patient.

## Supplementary Information

Supplementary Information.
