# Supplementary material for: Personalized risk predictor for acute cellular rejection in lung transplant using soluble CD31
Source: Sci Rep. 2022 Oct 21;12:17628. doi: 10.1038/s41598-022-21070-1 (PMC9587244; doi:10.1038/s41598-022-21070-1)
Supplement: Supplementary file 1 — Supplementary Information. [file 41598_2022_21070_MOESM1_ESM.docx]

**Supplemental material for**

**Personalised Risk Predictor for Acute Cellular Rejection in Lung transplant using soluble CD31**

Alexy Tran-Dinh*^1,2^ M.D, Ph.D; Quentin Laurent ^2^ M.D; Guillaume Even ^2^ BSc; Sébastien Tanaka ^1,3^ M.D Ph.D; Brice Lortat-Jacob M.D ^1^, Yves Castier ^4,5^ M.D, Ph.D; Hervé Mal ^5,6^ M.D, Ph.D; Jonathan Messika ^5,6,7^ M.D, Ph.D; Pierre Mordant ^4,5^ MD, PhD; Antonino Nicoletti ^2^ Ph.D, Philippe Montravers ^#1,5^ M.D, Ph.D; Giuseppina Caligiuri ^#2^ M.D, Ph.D; Ian Morilla* ^8^ Ph.D.

# These 2 co-authors contributed equally to this study

^1^ Université de Paris, AP-HP, Hôpital Bichat Claude Bernard, Département d’anesthésie-Réanimation, INSERM, Paris, France.

^2^ Université de Paris, LVTS, Inserm U1148, F-75018 Paris, France.

^3^ UMR 1188, INSERM, Université de la Réunion, Saint-Denis de la Réunion, France.

^4^ Université de Paris, AP-HP, Hôpital Bichat Claude Bernard, Département de chirurgie thoracique et vasculaire, Paris, France.

^5^ INSERM UMR 1152-ANR10-LABX-17.

^6^ Université de Paris, AP-HP, Hôpital Bichat Claude Bernard, Pneumologie B et Transplantation Pulmonaire, Paris, France.

^7^ Paris Transplant Group, Paris, France.

^8^ Université Sorbonne Paris Nord, LAGA, CNRS, UMR 7539, Laboratoire d'excellence Inflamex, F-93430, Villetaneuse, France.

Corresponding authors:

Ian Morilla: morilla@math.univ-paris13.fr

Alexy Tran-Dinh: alexy.trandinh@aphp.fr

In these notes, we provide complementary information useful to better understanding results shown in the main manuscript as well as to going deeper with the methods described in there. Data and the python codes used to analyse data and run the simulations of models (1 & 2) are available from the authors upon reasonable request.


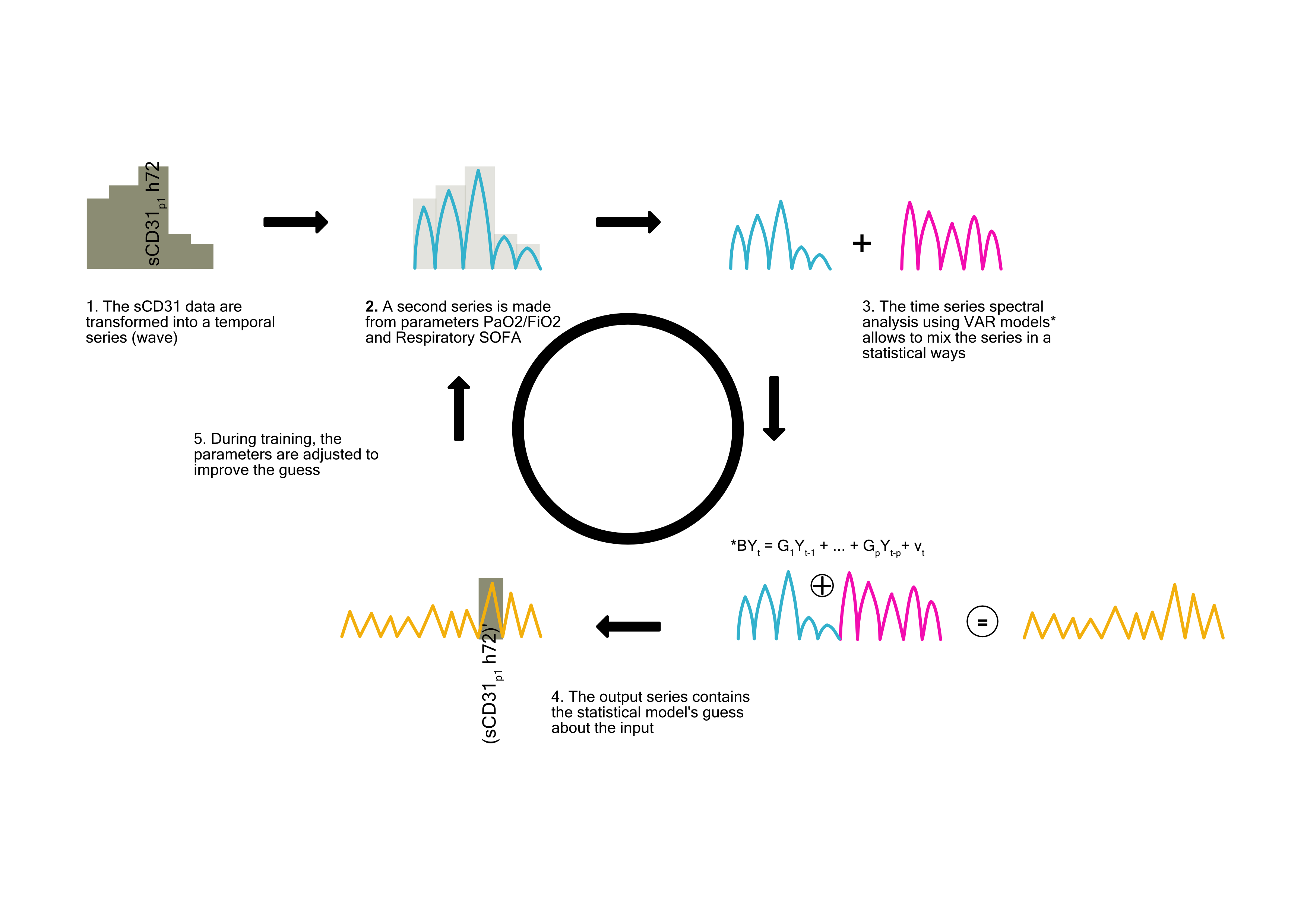


Figure S0. **Proposed time distributed and multimodal network model.** The sCD31 patient outcomes are transformed into time series (*ts*). A second series is made from important clinical indicators, i.e., PaO_2_/FiO_2_ and respiratory SOFA. Those *ts* are mixed by means of vector autoregressive (VAR) models and their spectral properties. The output from such mixture contains the VAR model’s guess about the input data. Finally, and in a feedback loop, we learn the best parameters that improve the model’s guess.

**Model 1.** Systematic evaluation of sCD31 as univariate time series.


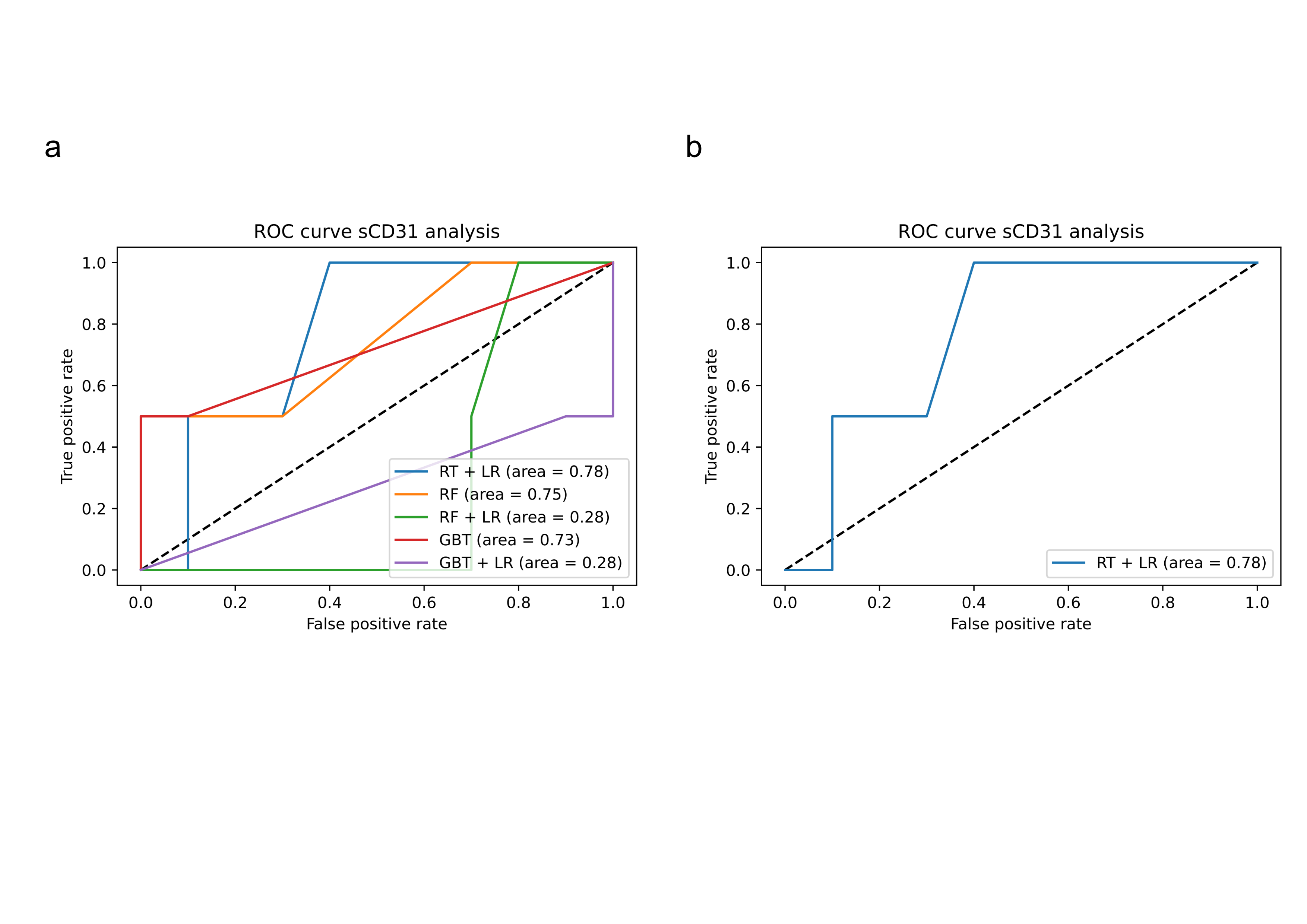
Figure S1. **Proposed time distributed network univariate.** ROC curves of the patient outcome prediction using sCD31 time series and multiples learning approaches. (a) Results on average during the first 24 hours, the first 48 hours, and after 72 hours. (b) Best models for the entire set of features. Initials in the inset stand for their auc results based on RT = Random Trees, LR = Logistic Regression (latent features space), RF = Random Forest, and Gradient Boosting Trees.

We picked 15 (out of 40 patients) estimators for the unsupervised/supervised learning. During the training, we split data with a test size equals 30% of total. For regression purposes, it is important to train the ensemble of trees on a different subset of the training data than the linear regression model to avoid overfitting, if the total number of leaves is similar to the number of training samples. Hence, we split the early test set into 50% as test size to training logistic regression. Such regression is worked out by means of the Large-scale Bound-constrained Optimization solver (*lbfgs*) leveraging a maximum of 1000 iterations. In particular, we passed through a OneHotEncoder the supervised transformation based on random forests and gradient boosted trees. Finally, we applied the method ‘predict_proba’ in module sklearn in all the prediction methods to calculi their false/true positive rates.

**Model 2.** Multivariate time distributed network.

**Table S1. Granger’s causation matrix.** The row is the rresponse (Y), and the columns are the predictor series (X). The values in the table are the P-Values. P-Values lesser than the significance level (0.05), implies the Null Hypothesis that the coefficients of the corresponding past values are zero, that is, the X does not cause Y can be rejected.

|  | sCD31_x | PaO_2_/FiO_2__x | Respiratory SOFA_x |
| --- | --- | --- | --- |
| sCD31_y | 1.0000 | 0.4005 | 0.2501 |
| PaO_2_/FiO_2__y | 0.0054 | 1.0000 | 0.0312 |
| Respiratory SOFA_y | 0.3036 | 0.0796 | 1.0000 |

The fundamental of Vector AutoRegression is that each of the time series in the system influences each other. In that way, we can predict the series based on past delays/lags of itself along its counterpart respect the other series in the system.

By using Granger’s causality test, it’s possible to check this relationship in advance to any model calculations. For example, the value 0.4005 in row 1 and column 2, it refers to the p-value of PaO_2_/FiO_2__x causing sCD31_y. Whereas, the 0.0054 in row 2 and column 1 refers to the p-value of sCD31_y causing PaO_2_/FiO_2__x.

Furthermore, cointegration test contributes to establish the presence of a statistically significant connection between two or more time series in the system. To digging deeper in such link, we first need to know what is the ‘order of integration’ (d).

Indeed, order of integration (d) is nothing, but the number of differencing required to a non-stationary time series becomes stationary.

Thus, the linear combination of two or more time series of order of integration (d) less than that of the individual series is said to be cointegrated.

Table S2. **Augmented Dickey-Fuller test.** Method based on Dickey-Fuller test to check the time series to be forecasted becomes stationary.

| **Augmented Dickey-Fuller Test on** | | |
| --- | --- | --- |
| "**sCD31**" | " **PaO_2_/FiO_2_**" | "**Respiratory SOFA**" |
| Null Hypothesis: Data has unit root. Non-Stationary. | | |
| Significance Level = 0.05 | Significance Level = 0.05 | Significance Level = 0.05 |
| Test Statistic = -8.0991 | Test Statistic = -6.5762 | Test Statistic = -6.5849 |
| No. Lags Chosen = 4 | No. Lags Chosen = 7 | No. Lags Chosen = 7 |
| Critical value 1% = -3.491 | Critical value 1% = -3.4393 | Critical value 1% = -3.493 |
| Critical value 5% = -2.888 | Critical value 5% = -2.889 | Critical value 5% = -2.889 |
| Critical value 10% = -2.581 | Critical value 10% = -2.581 | Critical value 10% = -2.581 |
| => P-Value = 0.0. Rejecting Null Hypothesis. | => P-Value = 0.0. Rejecting Null Hypothesis. | => P-Value = 0.0. Rejecting Null Hypothesis. |
| => Series is Stationary. | | |

Table S3. **VAR Order Selection (* highlights the minimums).** Since there is not a standard way of selecting the best indicator, we chose HQIC after comparing all the scores regarding the predictive power of the VAR model using their associated lag number.

|  | **AIC** | **BIC** | **FPE** | **HQIC** |
| --- | --- | --- | --- | --- |
| **0** | -1.196 | -1.119 | 0.3024 | -1.165 |
| **1** | -1.463 | -1.156* | 0.2317 | -1.338 |
| **2** | -1.523 | -0.9855 | 0.2183 | -1.487* |
| **3** | -1.605 | -0.8374 | 0.2013 | -1.294 |
| **4** | -1.676 | -0.6788 | 0.1878 | -1.272 |
| **5** | -1.696 | -0.4682 | 0.1848 | -1.199 |
| **6** | -1.729* | -0.2706 | 0.1798* | -1.138 |
| **7** | -1.632 | 0.05600 | 0.1994 | -0.9485 |
| **8** | -1.601 | 0.3173 | 0.2077 | -0.8241 |
| **9** | -1.551 | 0.5982 | 0.2213 | -0.6802 |
| **10** | -1.576 | 0.8030 | 0.2191 | -0.6123 |
| **11** | -1.522 | 1.087 | 0.2357 | -0.4655 |
| **12** | -1.503 | 1.337 | 0.2461 | -0.3526 |

Any VAR(L) model includes L lags of each variable in each equation. For example, for a two-variable system, each equation needs for 1+2L – Hence, we need 2(1+2L)=2+4L in total. Yet, for a general n-variable system, such a number increases until 1+kL for each equation. A general value would be n(1+2L)=n+2nL (https://www.ssc.wisc.edu/~bhansen/460/460Lecture25%202017.pdf). To select such a L, we selected HQIC after comparing all the scores regarding the predictive power of the VAR model using their associated lag number, as mentioned above.

To calculate information criterion for our models in the clinical estimators up to a maximum lag of maximum probability, we produced the convenient tables S4 and S6.

Table S4. **Summary of regression results.** VAR model by ordinary least squares (OLS) method with 3 equations. Particular metrics: BIC: -1.0547; Nobs: 112; HQIC: -1.4874; Log likelihood: -346.922; FPE: 0.1683; AIC: -1.7828; and Det (Omega_mle): 0.1302.

| Results for equation sCD31 | | | | |
| --- | --- | --- | --- | --- |
|  | coefficient | std. error | t-stat | prob |
| Const | 0.017505 | 0.086460 | 0.202 | 0.840 |
| L1.sCD31 | -0.444210 | 0.097192 | -4.570 | 0.000 |
| L1.PaO_2_/FiO_2_ | 0.009029 | 0.221111 | 0.041 | 0.967 |
| L1.respiratory SOFA | 0.016931 | 0.225687 | 0.075 | 0.940 |
| L2.sCD31 | -0.261284 | 0.103391 | -2.527 | 0.011 |
| L2. PaO_2_/FiO_2_ | 0.052219 | 0.237366 | 0.220 | 0.826 |
| L2.respiratory SOFA | 0.017258 | 0.245866 | 0.070 | 0.944 |
| L3.sCD31 | -0.002272 | 0.095397 | -0.024 | 0.981 |
| L3. PaO_2_/FiO_2_ | 0.333792 | 0.223506 | 1.493 | 0.135 |
| L3.respiratory SOFA | 0.412835 | 0.227662 | 1.813 | 0.070 |
| Results for equation PaO2/FiO2 | | | | |
| Const | 0.007897 | 0.097137 | 0.081 | 0.935 |
| L1.sCD31 | -0.103728 | 0.109195 | -0.950 | 0.342 |
| L1. PaO_2_/FiO_2_ | -0.483209 | 0.248418 | -1.945 | 0.052 |
| L1.respiratory SOFA | -0.064083 | 0.253558 | -0.253 | 0.800 |
| L2.sCD31 | -0.069502 | 0.116159 | -0.598 | 0.550 |
| L2. PaO_2_/FiO_2_ | -0.768934 | 0.266680 | -2.883 | 0.004 |
| L2.respiratory SOFA | -0.439339 | 0.276230 | -1.590 | 0.112 |
| L3.sCD31 | -0.195576 | 0.107178 | -1.825 | 0.068 |
| L3. PaO_2_/FiO_2_ | -0.291592 | 0.251109 | -1.161 | 0.246 |
| L3.respiratory SOFA | 0.080851 | 0.255777 | 0.316 | 0.752 |
| Results for equation respiratory SOFA | | | | |
| Const | -0.008002 | 0.096504 | -0.083 | 0.934 |
| L1.sCD31 | 0.058448 | 0.108484 | 0.539 | 0.590 |
| L1. PaO_2_/FiO_2_ | -0.157729 | 0.246800 | -0.639 | 0.523 |
| L1.respiratory SOFA | -0.548205 | 0.251907 | -2.176 | 0.030 |
| L2.sCD31 | 0.017832 | 0.115402 | 0.155 | 0.877 |
| L2. PaO_2_/FiO_2_ | 0.280424 | 0.264943 | 1.058 | 0.290 |
| L2.respiratory SOFA | -0.004380 | 0.274431 | -0.016 | 0.987 |
| L3.sCD31 | 0.167483 | 0.106480 | 1.573 | 0.116 |
| L3. PaO_2_/FiO_2_ | 0.054942 | 0.249473 | 0.220 | 0.826 |
| L3.respiratory SOFA | -0.289104 | 0.254112 | -1.138 | 0.255 |

Table S5. **Correlation matrix of residuals for the VAR regression results.** Additionally, the Durbin-Watson scores of the biomarker time series who capture potential overlooked pattern in the series remained unaltered (i.e., DW~2).

|  | sCD31 | PaO_2_/FiO_2_ | Respiratory SOFA | DW |
| --- | --- | --- | --- | --- |
| sCD31 | 1.000000 | 0.001146 | 0.028249 | 2.04 |
| PaO_2_/FiO_2_ | 0.001146 | 1.000000 | 0.052705 | 2.09 |
| RespiratorySOFA | 0.028249 | -0.925871 | 1.000000 | 2.06 |

Table S6. **Comprehensive set of metrics used to evaluate the forecasts.**

| Forecast Accuracy | sCD31 | PaO_2_/FiO_2_ | Respiratory SOFA |
| --- | --- | --- | --- |
| mape | 0.8231 | 0.6387 | 0.5638 |
| me | 0.4418 | 0.4085 | -0.9650 |
| mae | 0.5217 | 0.6927 | 0.9650 |
| mpe | -0.5975 | 0.0713 | -0.5638 |
| rmse | 0.7954 | 1.00139 | 1.1321 |
| corr | 0.1323 | 0.0006 | -0.0643 |
| minmax | 0.5179 | -1.1884 | 0.5638 |


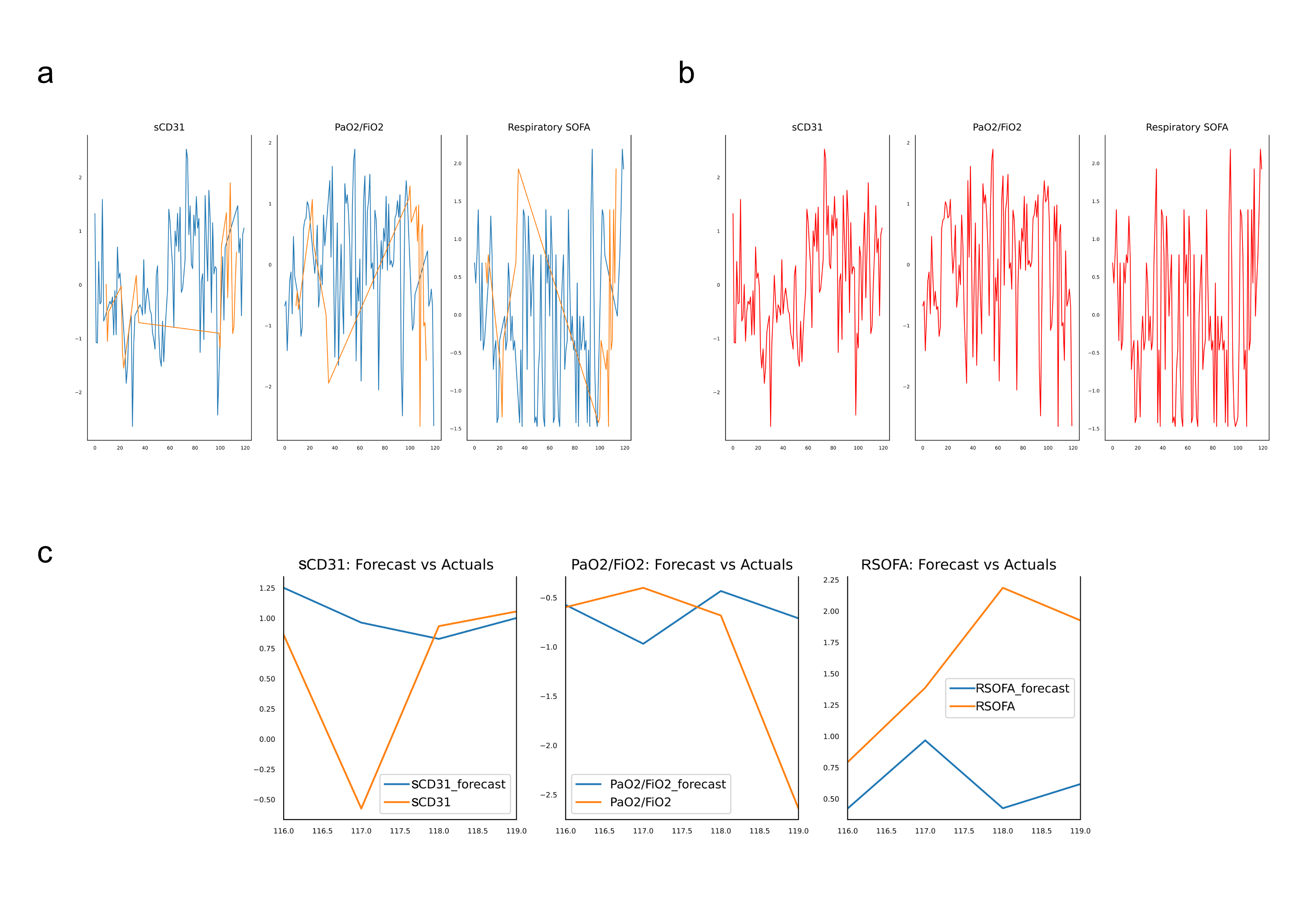


Figure S2. **Visualization of forecast vs actuals series.** (a) Time series representation of clinical features separated by Recovery (highlighted in blue) and ACR (in orange) classes. (b) Each of the series have a similar trend pattern over the time tags except for PaO_2_/FiO_2_, where an anti-correlated amplitude ranges the pattern. To prevent eventual artifact in the prediction, we fed it in a separate pipeline to our temporal deep network. (c) Predicted time series vs real highlighted in blue and orange respectively. The predictions exhibit all similar trends, which is the best indicator of our model’s goodness.


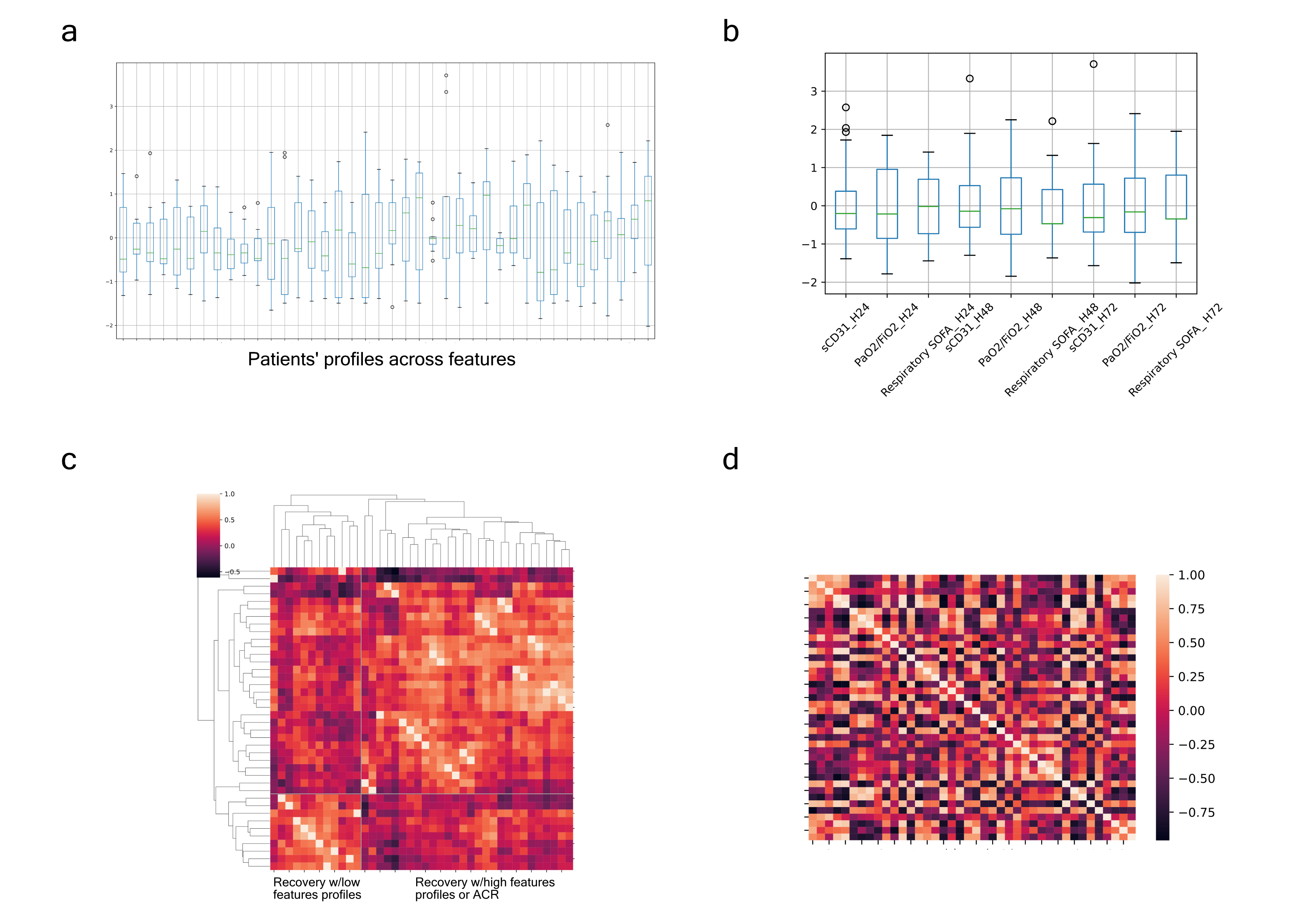


Figure S3. **Visualization of warping distance between time series of patient’s groups.** The distance function has linear space complexity but quadratic time complexity. (a) Profile of each patient associated with sCD31, PaO_2_/FiO_2_, and respiratory SOFA estimations upon log standardization. Barplots: Median, interquartile range, Q1+/-std and Q3+/-std along with outliers highlighted as small circles are shown. A flattened trend is observed, but for patients with high PaO_2_/FiO_2_ and respiratory SOFA estimations (>=400, >=3) or even labeled as ACR. (b) Same plot as (a) but showing how clinical features behave across patients. In that case, the regular behaviour is even more evident. What makes sure clinical features are fair to comparing patients’ outcomes during the post-transplantation monitoring. (c) Clustering of patients’ time series according to their *1-dtw* pairwise distances. The heatmap shows how patients treated as Recovery with a lower presence of sCD31, PaO_2_/FiO_2_, and respiratory SOFA grouped together. Likewise, those patients with a higher presence of clinical features or those catalogued as ACR. (d) Same figure as (c) but showing correlation between patients’ outcomes what enables distinguish between intra-clusters patients’ profiles. This demonstrates our VAR model can stratify patients early in the analysis. We use training, then, to improve such stratification.


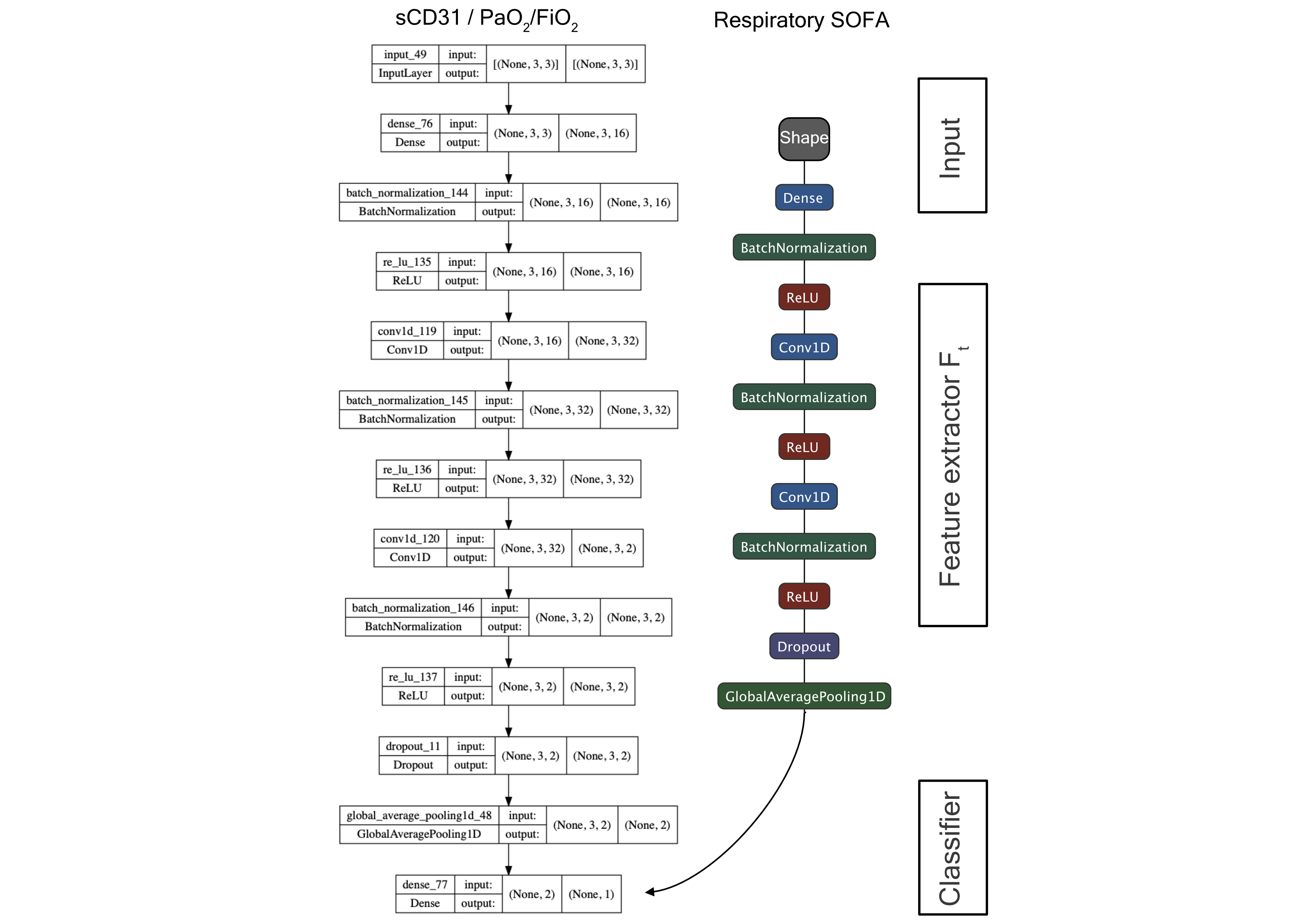


Figure S4. **Global** **architecture of our temporal network.** This architecture is composed by a linear filtering (see main manuscript) with a kernel with 16 units, two convolutional layers along batch normalization with 32 and 2 filters respectively, a flatten and dropout layers to prevent overfitting, and a final dense layer who is the actual classifier of the network. Additionally, we consider a parallel path for the respiratory SOFA series (i.e., coloured panels on the right-hand side of the main architecture) whose output is concatenated to feed a sigmoid classifier of the last dense layer.


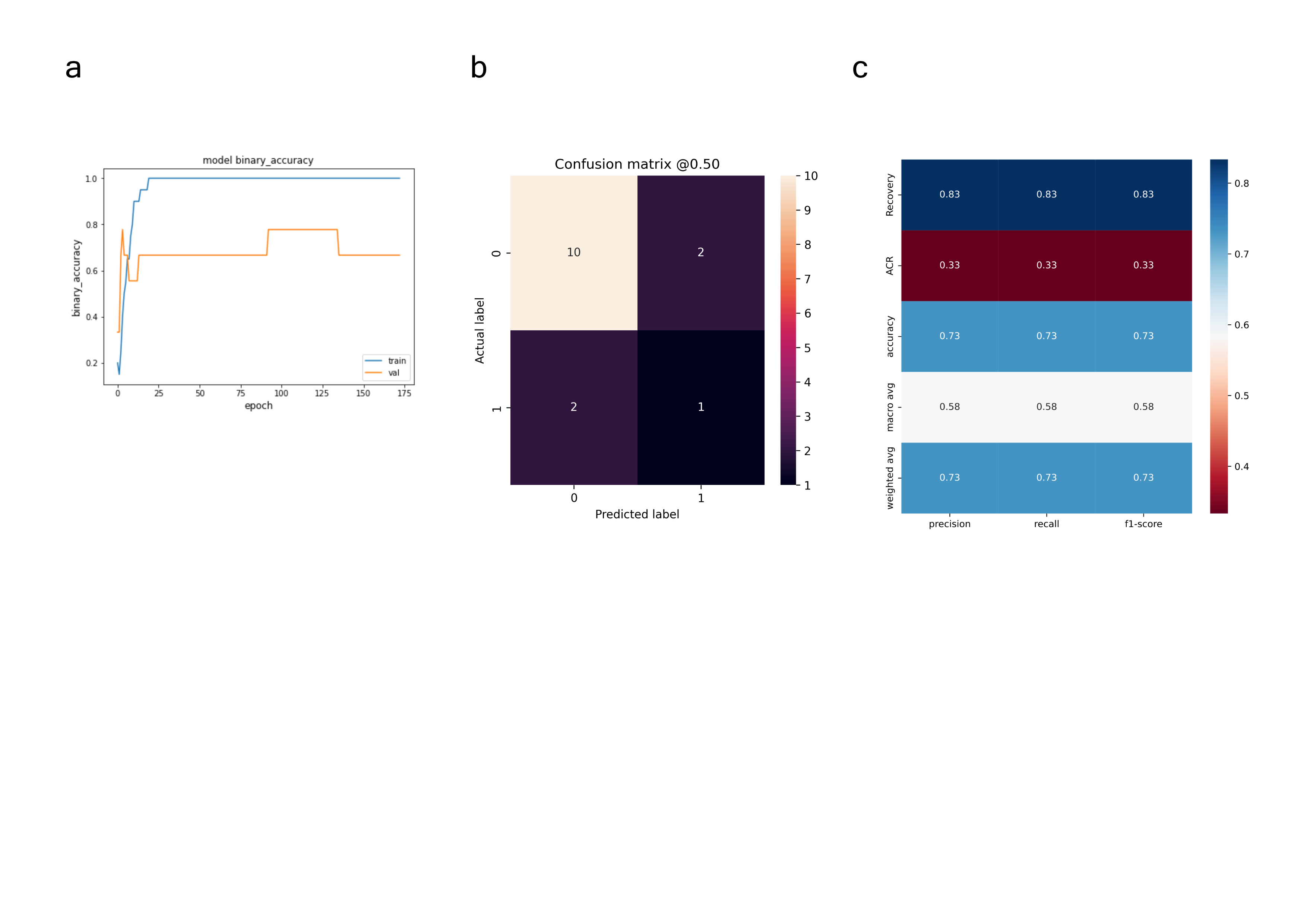
Figure S5. **Results from the baseline temporal network.** (a) Assessment of our model during testing validation of our baseline model. We plotted it in terms of roc curves. Validation’s curve is influenced by the dropout and global average pooling what makes it not getting the training curve closer by yielding a lower accuracy. (b) Goodness of our baseline deep network during the training task. We calculated its confusion matrix where we can observe four misclassified patients, but also the false negatives are less detected. (e) Final heatmap associated with the metrics used in the learning process of our baseline model. Accuracy is even good, but we might like to have higher recall and precision values to prevent missing false negatives.


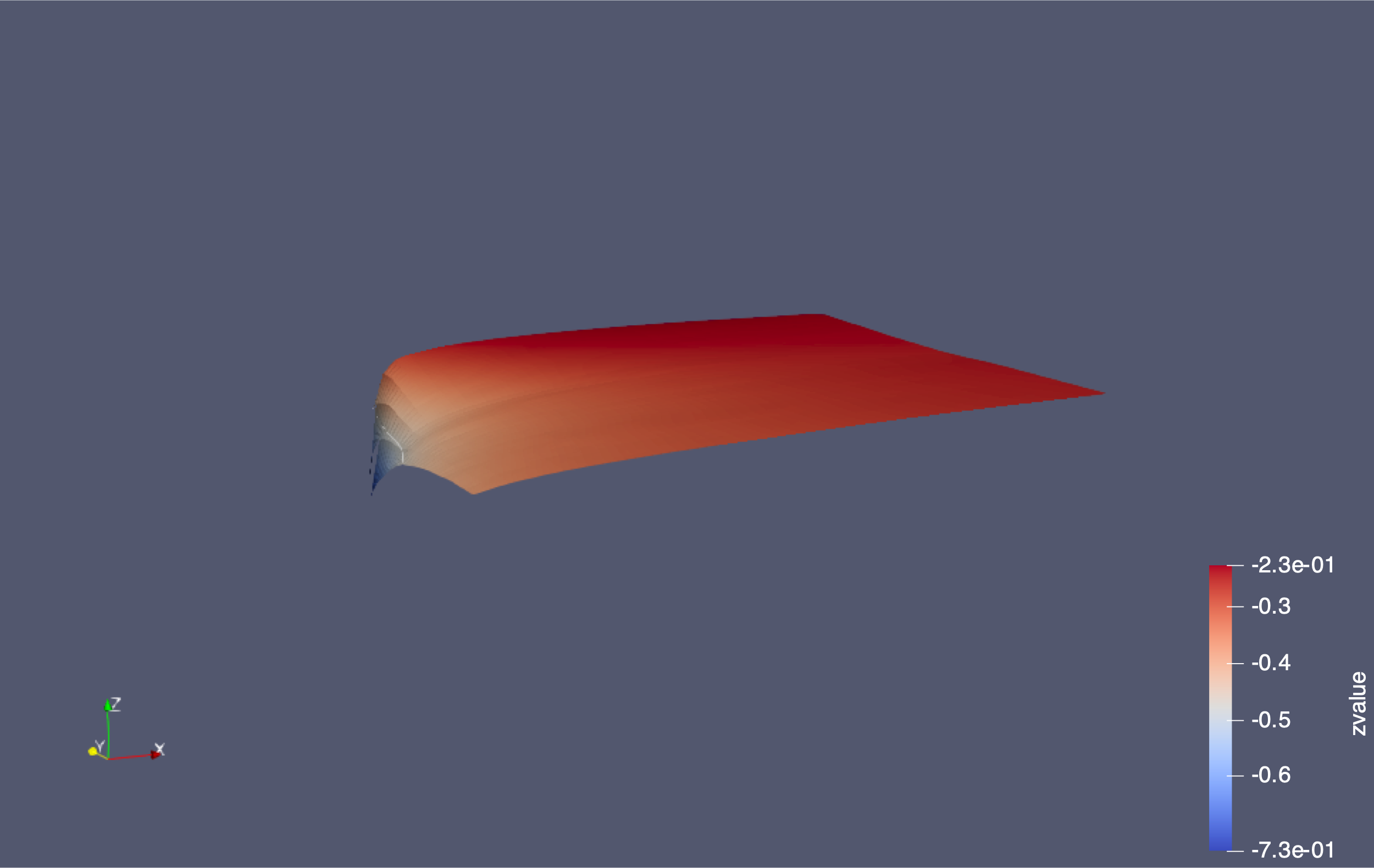


Figure S6. **Trainable parameter landscapes of the deep temporal network.** The plots show our model’s learning progression across epochs and batches. Grid projection exhibiting a global maximum collapsing in 1 and a stretcher option for “valleys”. Surface composed by the weights required to achieve the optimal sought by Adam algorithm, which is scaled by their z-scores. Contour with optimal projected trajectories in blue in the [-1, 1] range. Those trajectories go from “mountain” values nearby 1 to a “valley” nearby -0.73. From the green levels’ density and blue maps, we can confirm 1 and -0.73 as the global coordinates to be travelled by the network to arrive in good convergence.
